# A Framework for the Selection of Plant Growth-Promoting Rhizobacteria Based on Bacterial Competence Mechanisms

**DOI:** 10.1128/AEM.00760-20

**Published:** 2020-07-02

**Authors:** Carol V. Amaya-Gómez, Mario Porcel, Leyanis Mesa-Garriga, Martha I. Gómez-Álvarez

**Affiliations:** a Agricultural Microbiology Laboratory, La Libertad, Corporación Colombiana de Investigación Agropecuaria (AGROSAVIA), Villavicencio, Colombia; b La Libertad, Corporación Colombiana de Investigación Agropecuaria (AGROSAVIA), Villavicencio, Colombia; c Bioproducts Department, Tibaitatá, Corporación Colombiana de Investigación Agropecuaria (AGROSAVIA), Mosquera, Colombia; Shanghai Jiao Tong University

**Keywords:** bioproducts, plant growth-promoting rhizobacteria, *Rhizobium*, multicriteria decision analysis

## Abstract

The use of plant growth-promoting rhizobacteria (PGPR) is increasingly meaningful for the development of more environmentally friendly agricultural practices. However, often the PGPR strains selected in the laboratory fail to confer the expected beneficial effects when evaluated in plant experiments. Insufficient rhizosphere colonization is pointed out as one of the causes. With the aim of minimizing this inconsistency, we propose that besides studying plant growth promotion traits (PGP), the screening strategy should include evaluation of the microbial phenotypes required for colonization and persistence. As a model, we carried out this strategy in three *Rhizobium* sp. strains that showed phosphorus solubilization ability and production of siderophores. All strains displayed colonization phenotypes like surface spreading, resistance to hydrogen peroxide, and formed biofilms. Regarding their ability to persist, biofilm formation was observed to be influenced by pH and the phosphorus nutrient provided in the growth media. Differences in the competence of the strains to use several carbon substrates were also detected. As part of our framework, we compared the phenotypic characteristics of the strains in a quantitative manner. The data analysis was integrated using a multicriteria decision analysis (MCDA). All our results were scored, weighted, and grouped as relevant for PGP, colonization, or persistence. MCDA demonstrated that, when the phenotypes related to PGP and colonization are weighted over those for persistence, strain B02 performs better than the other two *Rhizobium* sp. strains. The use of our framework could assist the selection of more competent strains to be tested in greenhouse and field trials.

**IMPORTANCE** Numerous plant growth-promoting rhizobacteria (PGPR) have been inoculated into the soil with the aim of improving the supply of nutrients to crop plants and decreasing the requirement of chemical fertilizers. However, sometimes these microbes fail to competitively colonize the plant roots and rhizosphere. Hence, the plant growth promotion effect is not observed. Here, we describe a new screening strategy aiming at the selection of more competent PGPR. We evaluated bacterial phenotypes related to plant growth promotion, colonization, and persistence. Our results demonstrated that despite the fact that our *Rhizobium* sp. strains successfully solubilized phosphorus and produced siderophores, their abilities to spread over surfaces, resist hydrogen peroxide, and form biofilms varied. Additionally, a multicriteria decision analysis was used to analyze the data that originated from bacterial physiological characterizations. This analysis allowed us to innovatively evaluate each strain as a whole and compare the performances of the strains under hypothetical scenarios of bacterial-trait requirements.

## Abstract (precis)

Numerous plant growth-promoting rhizobacteria (PGPR) have been inoculated into the soil with the aim of improving the supply of nutrients to crop plants and decreasing the requirement of chemical fertilizers. However, sometimes these microbes fail to competitively colonize the plant roots and rhizosphere. Hence, the plant growth promotion effect is not observed. Here, we describe a new screening strategy aiming at the selection of more competent PGPR. We evaluated bacterial phenotypes related to plant growth promotion, colonization, and persistence. Our results demonstrated that despite the fact that our *Rhizobium* sp. strains successfully solubilized phosphorus and produced siderophores, their abilities to spread over surfaces, resist hydrogen peroxide, and form biofilms varied. Additionally, a multicriteria decision analysis was used to analyze the data that originated from bacterial physiological characterizations. This analysis allowed us to innovatively evaluate each strain as a whole and compare the performances of the strains under hypothetical scenarios of bacterial-trait requirements.

## INTRODUCTION

Plant growth-promoting rhizobacteria (PGPR) are used as biofertilizers. PGPR are able to enhance plant growth using several mechanisms that facilitate nutrient acquisition (1). The use of biofertilizers based on these microorganisms is a biological approach that has proven effective for increasing agricultural yield while contributing to good environmental practices (1–3). However, despite PGPR effectiveness in the laboratory, once these bacteria are tested under greenhouse conditions, or applied in field settings, the expected effects on plant development may not be observed (4, 5). Why this occurs remains a concern. One reason underlying this mismatch between laboratory and greenhouse and field trials could be that studies aiming at bioprospecting of PGPR have traditionally focused on plant growth promotion traits. Additionally, even though several microbial traits are commonly evaluated, the analysis of the results is rarely carried out in an integrated manner. Even when it is, phenotypes are classified as present or absent but not quantified.

Studies aimed at the screening of strains with plant growth-promoting abilities are generally initiated with the inoculation of serial dilutions of soil samples in nutrient-specific media. For instance, to select phosphorus solubilizers, National Botanical Research Institute’s phosphate (NBRIP) medium agar plates or liquid medium are employed (6). Change in color of bromothymol blue exhibits the alteration in the medium pH caused by the secretion of organic acids or phosphatases that allow phosphorus solubilization (7). Iron-chelating agents, such as siderophores, have also been recognized to participate in phosphorus solubilization (8). The detection of these molecules is carried out by growing microorganisms in media with low availability of iron and the chrome azurol S (CAS) assay (9). As a second step in screening strategies, greenhouse experiments are conducted with selected isolates. Parameters such as root length, plant dry matter weight, and plant productivity are normally evaluated (8). In this way, several strains of *Rhizobium*, *Pseudomonas*, *Bacillus*, *Enterobacter*, *Serratia*, *Paenibacillus*, *Azoarcus*, *Azospirillum*, and *Burkholderia*, among others, have been identified as plant growth promoters. Lastly, screening strategies evaluate the plant growth enhancement exerted by the most gifted strains in field trials.

In addition to plant growth bacterial traits, PGPR need to colonize the plant rhizosphere and root surface to enhance plant growth (1, 10, 11). Successful colonization will be achieved only if preceded by the detection of root exudates, movement of the microorganisms toward the plant roots, and biofilm formation over the roots or their surroundings (1, 10, 12–14). Chemotaxis is the known mechanism that microorganisms use for recognition of plant root exudates (10). The main types of bacterial motility described are swimming and surface spreading (15). Swimming is flagellum dependent and takes place in liquid environments. Detection of this type of motility is achieved in Bromfield medium (BM) containing 0.3% (wt/vol) agar (16). Surface spreading may be driven by flagella, type IV pili, focal adhesion complexes, and/or surfactant agents (15, 17). To study this kind of bacterial movement, it is necessary to provide nutritional requirements and reduce frictional forces on the agar surface. Thus, agar plates filled with different growth media containing 0.5 to 0.8% (wt/vol) agar concentrations are employed (17). Biofilm formation on the roots, as well as in the rhizosphere, facilitates nutrient exchange between the counterparts and direct supply of phytohormones and antimicrobial compounds (10, 18). Crystal violet staining of microbial biofilms formed on glass or polystyrene surfaces is the most commonly used method to evaluate this phenotype in bacteria (19). During the colonization stage, PGPR also need to overcome plant defense responses such as reactive oxygen species (ROS) secretion, a process in which antioxidant systems and polysaccharides are required (20).

Once the plant-bacterium interaction is initiated, in order to persist in the rhizosphere, PGPR rely on their capacity to survive biotic and abiotic stresses (1). At this stage of the interaction, biofilm formation is regarded as the main defense strategy against unpredictable and variable environmental conditions. Cells within the biofilm matrix have proven to be more resistant against antimicrobial compounds, desiccation, and UV light (10, 18). Additionally, nutrient scarcity is an innate soil characteristic that makes metabolic diversity essential for PGPR survival (21).

The evaluation of microbial phenotypes essential for the establishment of plant-bacterium interactions and bacterial performance described above is generally overlooked (1, 5, 22–24). Here, we propose that, together with the study of PGPR traits, during the first steps of the screening strategies for PGPR selection, the evaluation of bacterial mechanisms required for plant colonization and perdurance in the rhizosphere should be included. As a model, we evaluated the bacterial competence mechanisms of three *Rhizobium* strains selected for their ability to solubilize phosphorus and produce siderophores. We then exemplified the selection of strains for different purposes by using multicriteria analysis methodologies to gather our results and select the most promising strain under different scenarios.

## RESULTS

### Identification and evaluation of plant growth promotion traits.

In preliminary screening trials, several strains from AGROSAVIA’s microbial germplasm bank were evaluated for phosphate solubilization using NBRIP agar selective media. As B02, L3, and Sp20 excelled over others, they were selected for further studies. First, they were identified based on nucleotide sequence data. Comparisons of B02, L3, and Sp20 16S rRNA gene sequences with the NCBI GenBank database revealed similarities of 99%, 100%, and 99%, respectively, to reported *Rhizobium* sp. strains.

In the assays carried out in NBRIP agar plates containing Ca_3_PO_4_, FePO_4_, or rock phosphate as a phosphorus source, no significant differences were observed between the halo sizes formed by B02, L3, and Sp20 (linear models [LM]: *F *=* *0.15, df = 2, and *P = *0.858). The means of halo solubilization areas at 48 h postinoculation (hpi) were 1.5 cm^2^ for B02, 2.03 cm^2^ for L3, and 2.09 cm^2^ for Sp20. However, when the degrees of solubilization between phosphorus sources were compared independently of the strain, significant differences were observed between them (LM: Tukey’s test, *P < *0.050). Ca_3_PO_4_ was the most solubilized phosphorus nutrient (2.39 cm^2^), followed by rock phosphate (0.53 cm^2^) and FePO_4_ (0.43 cm^2^).

**FIG 1:**
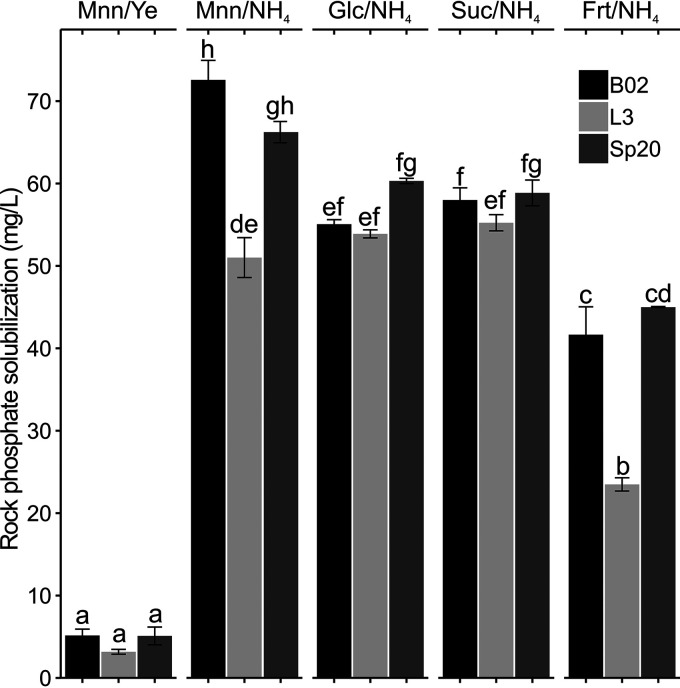
Mean (±SE) of phosphate solubilization ability in NBRIP broth (*n* = 3). Values followed by the same letter do no differ significantly (LM: Tukey’s test, *P < *0.001). NBRIP broth contained mannitol and yeast extract (Mnn/Ye), mannitol and NH_4_SO_4_ (Mnn/NH_4_), glucose and NH_4_SO_4_ (Glc/NH_4_), sucrose and NH_4_SO_4_ (Suc/NH_4_), and fructose and NH_4_SO_4_ (Frt/NH_4_).

In contrast to the assays carried out with NBRIP agar plates, the evaluation of phosphate solubilization in liquid media showed significant differences between the abilities of B02, L3, and Sp20 to solubilize rock phosphate (Fig. 1). Very low phosphate solubilization was detected in the broth containing yeast extract and mannitol (Mnn/Ye). However, replacement of yeast extract with NH_4_SO_4_ was enough to produce an increase in the solubilization of this nutrient in all the strains (Fig. 1). Our results demonstrate that phosphorus solubilization is also affected by the carbon source present in the medium when NH_4_SO_4_ is used as a nitrogen source. Remarkably, B02 displayed the highest solubilization of rock phosphate in Mnn/NH_4_, solubilizing 72.6 mg/liter. In addition to phosphate solubilization, we followed bacterial growth and pH drop in each broth (see Table S2 in the supplemental material). We observed that bacterial growth was not directly related to phosphate solubilization (Table S2). Regarding pH, B02 showed the most pronounced pH decline when grown in Mnn/NH_4_. A drop of 1.86 U was detected.

The loss of the CAS solution blue dye demonstrated that B02, L3, and Sp20 produced siderophores. A total of 86.1% of the iron present in the solution was captured by B02. L3 and Sp20 were able to capture 88.4% and 59.4%, respectively. B02 and L3 captured 28% more iron from the CAS-Fe(III) complex and significantly differed from Sp20 (LM: Tukey’s test, *P < *0.050).

### Study of bacterial colonization and persistence mechanisms.

**FIG 2:**
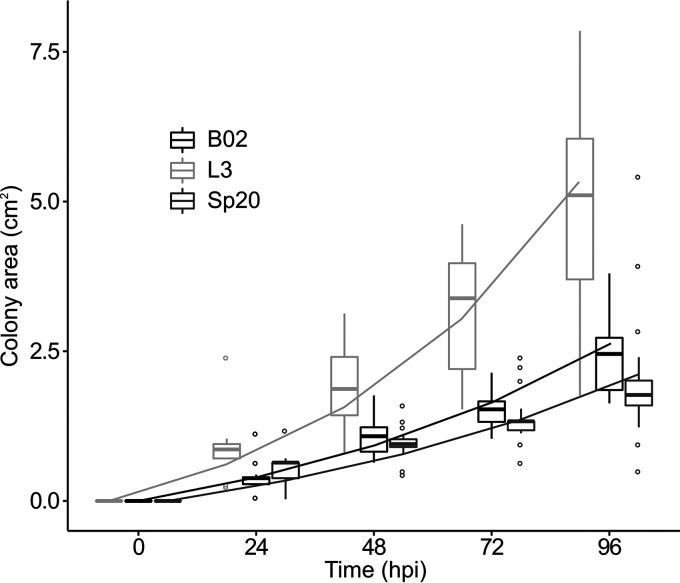
Boxplot of surface spreading of B02, L3, and Sp20 on semisolid modified MM (MMS). Boxes represent the 1st to 3rd interquartile ranges. The lines within the boxes indicate the median, the whiskers represent quartiles ± 1.5× interquartile distance, and the circles indicate outliers. Lines represent the predicted values of the LMM. Values for L3 differed significantly from those for the other strains (LMM: Tukey’s test, *P < *0.050).

**(i) Swimming motility and surface spreading.** Swimming assays revealed that B02, L3, and Sp20 are motile. Nonetheless, no significant differences were obtained between our strains when swimming halo areas were analyzed (data not shown). With respect to surface motility, we observed different patterns and halo sizes of the spreading colonies. B02 and Sp20 colonies had the shape of a drop that expands on the surface of the agar plates. The L3 colony resembled a star shape (Fig. S1). As shown in Fig. 2, measurements of spreading colony areas at 24, 48, 72 and 96 hpi demonstrated that L3 excelled in this phenotype over B02 and Sp20 (linear mixed-effects models [LMM]: Tukey’s test, *P < *0.005 [Fig. 2]).

**FIG 3:**
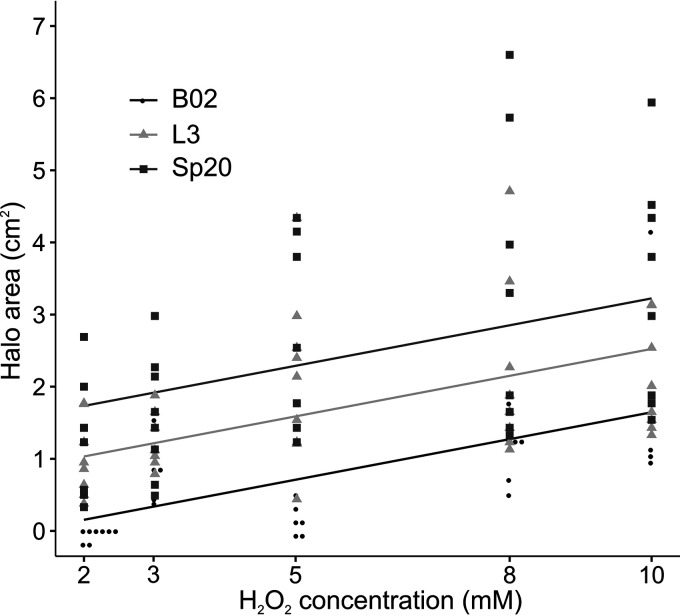
Sensitivity to hydrogen peroxide. Lines represent the predicted values of the LM. Statistical differences were found between the three strains tested (LM: Tukey’s test, *P < *0.050).

**(ii) Resistance to reactive oxygen species.** To evaluate the capacities of B02, L3, and Sp20 to overcome reactive oxygen species, their sensitivities to different hydrogen peroxide concentrations was tested in MM. Analysis of the sensitivity halo areas revealed significant differences in resistance to hydrogen peroxide concentrations between the three strains (LM: Tukey’s test, *P < *0.050 [Fig. 3]). B02 was more resistant than L3 and Sp20 to all tested H_2_O_2_ concentrations.

**(iii) Biofilm formation ability.** The ability of our *Rhizobium* strains to developed biofilms was studied under the conditions shown in Table 1. As indicated by crystal violet staining, B02, L3, and Sp20 developed biofilms on glass surfaces under all evaluated conditions (Table 1). Compared to biofilms formed in minimal medium (MM) modified and supplemented with soil extract (MMS), acidic pH and presence of rock phosphate led to a decrease in the biofilms formed by the three strains. A temperature increase from 28°C to 35°C caused biofilm reduction only in B02. Biofilm formation under these conditions was not affected in L3 or Sp20.

**TABLE 1:** Biofilm formation on glass surface^*a*^

Biofilm formation condition	Absorbance (mean ± SE) for indicated strain		
	B02	L3	Sp20
Control (MMS)	0.46 ± 0.03 AB	0.53 ± 0.03 AB	0.54 ± 0.04 AB
Acidic pH	0.22 ± 0.01 C	0.21 ± 0.01 C	0.22 ± 0.02 C
Phosphorus fertilizer	0.22 ± 0.02 C	0.24 ± 0.02 C	0.23 ± 0.02 C
High temperature	0.18 ± 0.01 C	0.58 ± 0.02 B	0.42 ± 0.04 A

a Means (± SE) of absorbance at 550 nm are presented. Different letters indicate statistically significant differences (LM: Tukey’s test, *P *<* *0.050). Biofilm formation was assessed in modified minimal medium (MMS), in MMS with pH of 5.5 (acidic pH), in MMS containing rock phosphate (phosphorus fertilizer), and in MMS incubated at 35°C (high temperature).

**FIG 4:**
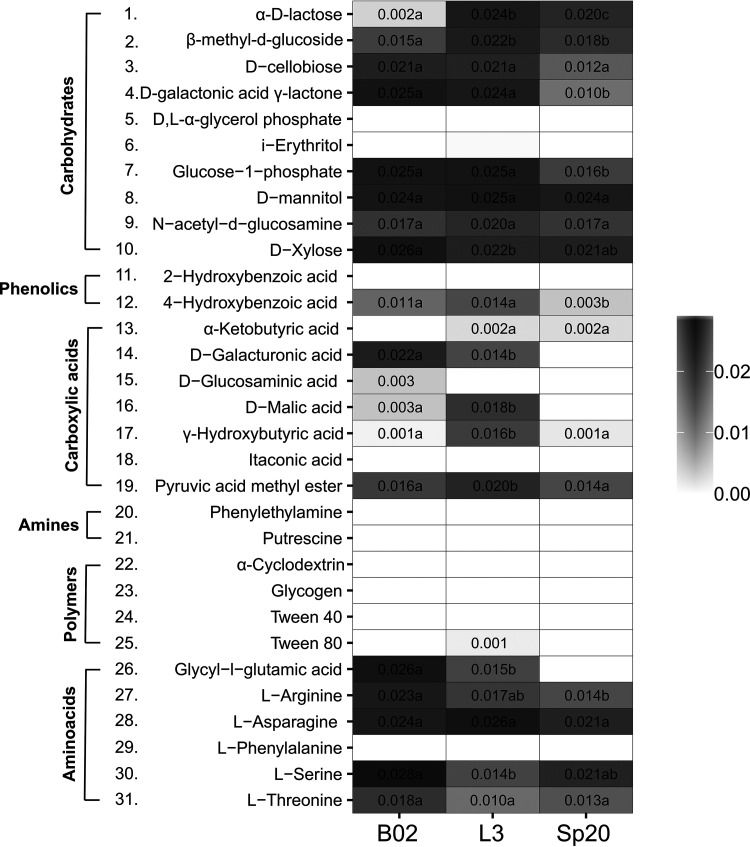
Metabolic fingerprint. Degradation velocities (increase in optical density per hour) are presented. Different letters represent statistically significant differences for degradation velocities between strains (LMM: Tukey’s test, *P < *0.050).

**(iv) Metabolic diversity.** The analysis of sugar degradation in Biolog Eco plates demonstrated that B02, L3, and Sp20 differed in their metabolic fingerprints. A heat map shows the similarities and differences between velocities of carbon source degradation for our three strains (Fig. 4). L3 is the strain with the highest metabolic diversity. It was able to use 17 out of the 31 carbon sources. It was followed by B02, which metabolized 15 substrates, and Sp20, which was able to use 13 (Fig. 4). The three strains were similar in the inability to metabolize 13 out of the 31 carbon sources. They were unable to metabolize any of the amines and polymers. According to our results, d-mannitol, *N*-acetyl-d-glucosamine, d-xylose, and l-asparagine are the carbon sources that are most easily assimilated by B02, L3, and Sp20 (Fig. 4). The most remarkable differences in degradation velocity between strains occurred for d-lactose and glycyl-l-glutamic acid.

### (v) Multicriteria decision analysis (MCDA).

**FIG 5:**
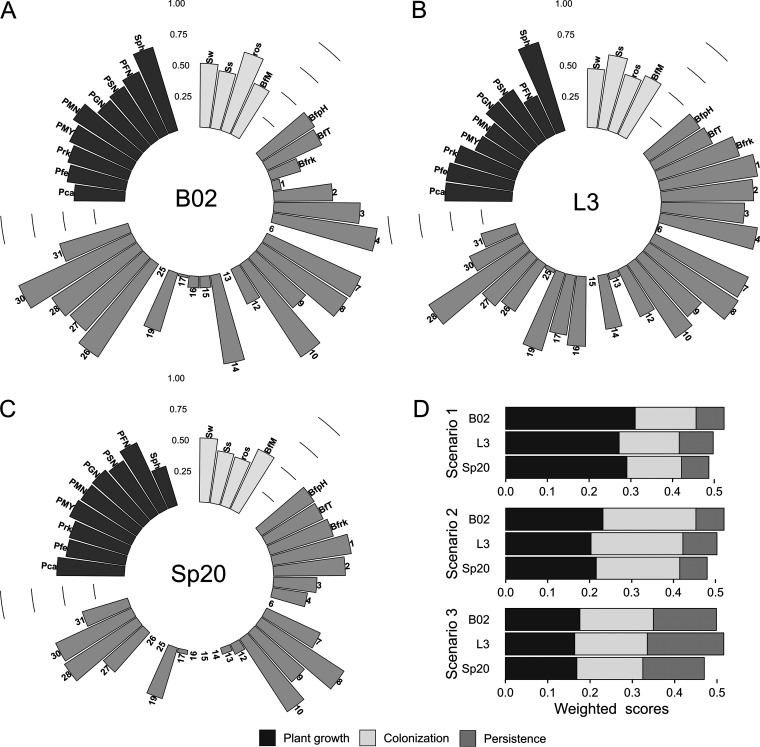
Multicriteria decision analysis. Panels A, B and C show attribute scores obtained for B02, L3, and Sp20. Attributes are as follows: phosphate solubilization in NBRIP containing Ca_3_PO_4_ (Pca), FePO_4_ (Pfe), rock phosphate (Prk), mannitol and yeast extract (PMY), and NH_4_SO_4_ and mannitol (PMN), glucose (PGN), sucrose (PSN), or fructose (PFN); siderophore synthesis (Sph), swimming (Sw), surface spreading (Ss), ROS sensitivity (ros); biofilm formation in modified minimal medium (BfM), at acidic pH (BfpH), at high temperature (BfT), and in the presence of rock phosphate (Bfrk); and utilization of α-d-lactose (1), β-methyl-d-glucoside (2), d-cellobiose (3), d-galactonic acid γ-lactone (4), i-erythritol (6), glucose-1-phosphate (7), d-mannitol (8), *N*-acetyl-d-glucosamine (9), d-xylose (10), 4-hydroxybenzoic acid (12), α-ketobutyric acid (13), d-galacturonic acid (14), d-glucosaminic acid (15), d-malic acid (16), γ-hydroxybutyric acid (17), pyruvic acid methyl ester (18), Tween 80 (25), glycyl-l-glutamic acid (26), l-arginine (27), l-asparagine (28), l-serine (30), and l-threonine (31). D, total weighted scores and weighted scores for each criterion under different scenarios. Weighted scores for each attribute are presented in Table S2.

The three bacteria presented considerable differences in unweighted scores for the different attributes considered (Fig. 5A to C), and particularly in the attributes related to persistence in the rhizosphere. Bacterium ranking based on weighted total scores (Fig. 5D) revealed that strain B02 excelled over L3 and SP20 under scenarios 1 and 2, where plant growth and colonization-related phenotypes were assigned higher scores. However, for scenario 3, the ranking shifted toward L3. In this case, equal scores were assigned to the three groups.

## DISCUSSION

Competitive colonization of plant roots and the rhizosphere by PGPR is imperative to aid nutrient acquisition in plants. Most screening strategies carried out to select plant growth promoters do not consider the study of the phenotypes required for bacterium-plant interaction establishment and persistence in the rhizosphere. In this work, we demonstrate that three *Rhizobium* strains that solubilize phosphorus and produce siderophores significantly differed in several of the phenotypes required for colonization of plant root surface and endurance in the rhizosphere. Given that the performance in the field of these microbes depends on several traits, we propose that they need to be considered and quantitatively compared to select the most competent strains.

The insight we obtained about the phosphorus solubilization ability of our strains varied according to the technique employed. Evaluation of phosphorus solubilization in solid NBRIP media did not reveal differences between the abilities of B02, L3, and Sp20 to solubilize the different phosphate sources. However, evaluation of this phenotype in liquid media showed that B02 performed better in this regard than L3 and Sp20. As Bashan et al. (23) proposed, our results highlight that adequate selection of phosphate-solubilizing strains requires the assessment of this ability in both solid and liquid media. Phosphorus solubilization in the NBRIP broth was also found to depend on the nitrogen and carbon sources present (25–27). As observed for B02, L3, and Sp20, Kshetri et al. (28) obtained higher solubilization in rhizobia when diammonium sulfate was used as a nitrogen source. In the presence of ammonium salts, the liberation of phosphate from minerals has been correlated with proton excretion during ammonium assimilation (29). Production of organic acids and production of siderophores have also been described as bacterial mechanisms involved in phosphorus solubilization (8). In both cases, phosphorus availability could be increased via metal chelation (30). Further studies are needed to gain a deeper understanding of the mechanisms used by our strains to solubilize phosphorus.

Following application in the field, bacteria sense root exudates and mucilage. Through chemotaxis and motility, they are able to orient their movements and approach the root surface (17, 31). Nonmotile strains of PGPR display lower survival averages. They have also been found to be less efficient in the colonization of plant roots (12, 32). Despite the importance of motility, this bacterial trait is rarely taken in consideration when selecting PGPR. In some studies, swimming motility is evaluated to determine whether the bacteria are motile (33). In the case of surface spreading, it is evaluated only in studies that aim to get a deeper understanding of the environmental and genetic control determinants involved (34, 35). These studies demonstrate that there are common factors like surface contact, nutrient depletion, or agar concentration in the media that are associated in the regulation of this phenotype among surface-motile bacteria (17). Therefore, general protocols that take into account these prevailing regulation mechanisms could be applied to study surface spreading during PGPR screenings (17, 36). Modifications made to the protocol described by Soto et al. (37) allowed us to observe surface spreading in B02, L3, and Sp20. Further research is required to verify whether the modifications made to this protocol contribute to reveal surface spreading in soil bacteria of different taxa.

During the first minutes of interaction, plants activate an induced systemic resistance response. This response is activated regardless of the beneficial or pathogenic nature of the bacteria (38, 39). Activation of the signal networks triggers chemical defenses, like the bursting of reactive oxygen species (ROS) (38, 40). To combat ROS, bacteria have antioxidant systems such as production of catalase and superoxide dismutase enzymes (38). These enzymes keep ROS at an equilibrium in the cells. Strains that present a weakened resistance to ROS are unable to establish bacterium-plant interactions (38). Hence, to be able to select competent PGPR strains with efficient antioxidant systems, the evaluation of this phenotype needs to be included. The methodology used to test B02, L3, and Sp20 for this aspect has been widely employed. It facilitates, in an indirect manner, the fast measurement of the strain’s ROS-scavenging ability. This methodology could additionally be used to test bacterial resistance to other biological or chemical compounds.

Even though PGPR may successfully colonize the plant root surface and its vicinity initially, their perdurance over time is not ensured. In this regard, both for the initial stages and for the persistence of the bacterium-plant interaction, biofilm formation is considered a key bacterial attribute. Persistence of a high population density attached to the root surface facilitates the integration of host and self-derived signals (18). The production and release of these compounds are synchronized to enhance plant growth. Once organized as biofilm, the PGPR are able to coordinate better to confront indigenous microbes. Furthermore, biofilms act as a protective barrier from ROS and other environmental stresses (41). Cells within a biofilm are more resistant to fluctuating conditions in soil, to antimicrobials excreted by the other soil inhabitants, and to predation. In most of the studies in which biofilm formation is evaluated, this phenotype is characterized in the selected strain during the final stage of the screening procedure (41). Colony counts of the attached cells or fluorescence-marked strains make it possible to visualize the biofilms formed on the root surface. Considering the importance of this phenotype, we propose to evaluate biofilm formation ability in all the promising strains. Crystal violet staining of biofilms developed on multiwell microtiter plates or laboratory glass tubes make it easier to study this phenotype. Using these methodologies, it is possible to study biofilm formation on several strains and under contrasting environmental and nutritional conditions that could impact PGPR performance in the field (19).

Strigul and Kravchenko (5) developed a mathematical model to predict the impact of factors affecting the performance of microbial inoculants in the field. Simulation outcomes showed that among the evaluated factors, the competition for scarce nutrients was decisive for PGPR survival. So, another crucial factor for persistence of PGPR in the plants rhizosphere would be their ability to adapt to nutrient scarcity (21). Plant growth-promoting strains with higher metabolic diversity would be able to adjust more easily to changes in nutrient availability. They would be better equipped to use soil nutrients whenever they became available. By readapting their metabolism, they will be able to continue to proliferate. Biolog Eco plates are primarily used for soil community analysis based on sole-carbon-source utilization (42). In our study, these plates facilitated the identification of the strain with higher metabolic diversity.

As far as we know, this is the first time that results obtained from the phenotypic characterization of PGPR have been integrated using a structured method. Decision-making analysis to select PGPR strains for bioproduct development requires exploration of all the results obtained in an integrated manner. Multicriteria decision-making analysis offers a systematic way to consolidate information from different inputs as well as an organized method to compare and categorize alternative scenarios (43). In our case, the sum of the individual presence or absence of the phenotypes displayed by each strain would not be enough to select the most promising candidate. They all demonstrated abilities in phosphate solubilization, motility, resistance to ROS, biofilm formation, and metabolic diversity to different extents. Using MCDA we were able to identify that in at least two of our scenarios, strain B02 could be the strain that would potentially perform better in the field than L3 or Sp20. Nonetheless, greenhouse and field trials evaluating the performance of B02 need to be carried out to corroborate this hypothesis.

Ultimately, in order to be operative when applied in the field, PGPR rely upon the phenotypes that allow colonization and persistence in the rhizosphere. A successful biofertilizer needs to be able to compete with indigenous microbes and capable of overcoming potentially harsh abiotic conditions. Notwithstanding the differences between the abilities of PGPR to promote plant growth, they need to be able to reach the roots (motility), overcome plant defense (ROS burst), and colonize the root surface through biofilm formation (1). Bacterial motility and biofilm formation were considered in our study because research with model PGPR belonging to the *Pseudomonas*, *Bacillus*, and *Rhizobium* genera has demonstrated that mutants with changes in these microbial traits are less efficient in root colonization and plant-bacterium interaction establishment (1, 12, 44, 45). Resistance to reactive oxygen species burst has also been found to be critical for successful colonization (38, 46). Regardless of the nature of the microorganisms, beneficial or pathogenic, the plant systemic response is activated at an early stage of the bacterium-plant interaction. PGPR require enzymatic and nonenzymatic antioxidant mechanisms to overcome this response. There are several microbial traits that were not considered in our research. We propose that depending on the future prospect of the bioproduct, the screening strategy should be improved by including the study of other relevant microbial phenotypes. Following the key requirements for a PGPR to be used in a biofertilizer as described by Finkel et al. (3) and Lugtenberg and Kamilova (1), we considered it appropiate to group the evaluated phenotypes as relevant for plant growth promotion, colonization, or persistance in the rhizosphere. However, MCDA allows the comparison, including any possible number of categories that can be defined based on the priorities considered relevant for the development of a specific bioproduct.

MCDA for PGPR-based bioproduct development could be boosted by including the results of other PGPR attributes. Plant growth promotion profiles of the candidate strains could be better understood if the ability of the isolates to fix nitrogen or to produce phytohormones is also evaluated (10). The study of other competence mechanisms such as chemotaxis, growth rate, or production of antimicrobial compounds could be included, too. One of the major advantages of MCDA is that it allows the integration and quantitative analysis of different types of data. Thus, not only data obtained in the laboratory but also results derived from greenhouse experiments and field trials could be integrated as part of the characterization setting. For whole-genome-sequenced strains, the presence or absence of known gene sequences of interest could be also considered. Additionally, analyzing the compatibility of several strains could facilitate the selection of microorganisms to be mixed in consortium-based bioproducts.

### Conclusion.

Nearly all microbes are forced to fight for resources against their counterparts that inhabit the same niche. The number of microbial competitors is usually vast, and therefore, a wide range of mechanisms are required in order to survive. PGPR applied as biofertilizers require all the competitive mechanisms they rely on to establish plant-bacterium interaction and prevail in the rhizosphere. Physiological characterization of the three *Rhizobium* sp. strains of our study exhibited how different they are in their plant growth promotion and competence abilities. They were all able to move, survive different concentrations of H_2_O_2_, form biofilms, and metabolize different carbon substrates. Simplifying selection of the most competent strain by sum of the phenotypes that they exhibited was not possible. Thus, MCDA facilitated the integration and quantitative analysis of our results, assisting selection of the most promising strain. It also offered the possibility to consider different scenarios to evaluate what would happen in the case that some phenotypes were more desirable than others. Strain selection following MCDA methodologies could benefit from a set of normalized weights, established for specific phenotype requirements, e.g., particular soil properties, specific crops, or climatic conditions. Testing the performance of PGPR strains in field trials would contribute to such a purpose. More research is required to better predict the relevance and the score assigned to each of the mechanisms studied.

## MATERIALS AND METHODS

### Bacterial strains and culture conditions.

Strains B02, L3, and SP20 were obtained from AGROSAVIA’s microbial germplasm bank. Routinely, they were grown in tryptone-yeast extract complex agar or broth (TY [50]). Unless otherwise indicated, microorganisms were grown at 28°C. Genomic DNA of B02, L3, and Sp20 was isolated from broth cultures using a PowerSoil kit (MoBio, Carlsbad, CA) following the manufacturer’s instructions. The partial sequence of the 16S rRNA gene was amplified using primers 518F (5′-CCAGCAGCCGCGGTAATACG-3′) and 800R (5′-TACCAGGGTATCTAATCC-3′). DNA sequencing was performed by Corpogen Corporation (Bogotá, Colombia). Strain identification to the genus level was carried out using the NCBI GenBank database and a basic local alignment search analysis tool (BLASTn).

### Phosphate solubilization assays.

Phosphate solubilization was first evaluated by measuring the halo solubilization areas formed by B02, L3, and Sp20 in the National Botanical Research Institute’s phosphate (NBRIP) medium, prepared as described by Nautiyal (6). NBRIP medium was supplemented with 1 g of Ca_3_(PO_4_)_2_, FePO_4_, or rock phosphate. Medium pH was adjusted to 7 after autoclaving. The plates were inoculated with drops of 3 μl of inoculum at a concentration of 5 × 10^8^ CFU/ml. The diameters of the halos were measured 24 and 48 h postinoculation (hpi). We also studied phosphate solubilization activity in different liquid media in which the carbon or nitrogen source was modified. B02, L3, and Sp20 were grown in mannitol-yeast extract (Mnn/Ye), mannitol-NH_4_SO_4_ (Mnn/NH_4_), glucose-NH_4_SO_4_ (Glc/NH_4_), sucrose-NH_4_SO_4_ (Suc/NH_4_), and fructose-NH_4_SO_4_ (Frt/NH_4_) broths containing 5 g of rock phosphate. Details of the medium compositions are described in Table S1. A total of 45 ml of each of these media was inoculated with 5 ml of an inoculum at a concentration of 1.2 × 10^9^ CFU/ml. After 24 h of fermentation, the cell supernatant was recovered to determine soluble phosphate. In this case, phosphate solubilization was evaluated by a photometric phosphate test (Merck, Darmstadt, Germany). The final concentration of cells (CFU per milliliter) and pH of the culture were also measured (Table S2).

### Siderophore detection by CAS assay.

Bacteria were grown in an iron-deficient medium (IDM) (9) to an optical density (OD) of 1.2. Siderophores production was evaluated as described by Schwyn and Neilands (9) using the chrome azurol S (CAS) liquid assay. The amount of iron removed from original blue CAS-Fe(III) complex was calculated by measuring absorbance at 630 nm. The spectrophotometer absorbance was set to zero using IDM treated with the CAS solution. The percentage of siderophore units was estimated by multiplying by 100 the absorbance measurement obtained per sample.

### Swimming motility and surface spreading.

Swimming motility was evaluated in Bromfield medium (BM) (16) containing 0.3% agar. Plates were inoculated using 3 μl of an overnight (ON) culture of B02, L3, and Sp20 grown in TY (5 × 10^8^ CFU/ml) and incubated for 48 h. Swimming colony diameter was measured at 24 and 48 hpi. Surface spreading was evaluated as described by Nogales et al. (35) in minimal medium (MM) (47) modified and supplemented with soil extract (MMS) as follows: glutamate (0.65 mM), mannitol (0.55 mM), mineral salts (K_2_HPO_4_, 0.13 mM; KH_2_PO_4_·3H_2_O, 0.22 mM; MgSO_4_·7H_2_O, 0.06 mM; CaCl_2_·2H_2_O, 0.034 mM; FeCl_3_·6H_2_O, 0.0022 mM; and NaCl, 0.086 mM) and agarose (0.6%). MMS was supplemented with vitamins (biotin, 0.2 mg/liter, and calcium pantothenate, 0.1 mg/liter) and 50 ml of soil extract obtained as described by Olsen and Bakken (48). B02, L3, and Sp20 were grown in TY medium and concentrated in MMS to a final concentration of 1 × 10^10^ CFU/ml. Surface spreading plates were inoculated using 2-μl droplets of the concentrated cell suspension. Spreading colony diameters were measured 24, 48, 72, and 96 hpi.

### Resistance to reactive oxygen species.

One-hundred-microliter volumes of overnight cultures of B02, L3, and Sp20 were added to 5 ml of semisolid minimal medium (SSMM) (47) containing 0.6% agar and poured over 20 ml of solid MM (1.3% agar). Three-millimeter-diameter paper discs were saturated with H_2_O_2_ at final concentrations of 2 mm, 3 mm, 5 mm, 8 mm, and 10 mm. Then the paper discs were placed on the top of the SSMM. The diameter of the growth-inhibitory halo was measured at 48 hpi.

### Biofilm formation assay.

Biofilm formation was evaluated under four different conditions (Table 2). Three-milliliter volumes of MMS broth, with modified pH or phosphorus sources as described in Table 1, were inoculated with 30 μl of a cell suspension containing 5 × 10^8^ CFU/ml. Tubes were incubated at 28°C for 48 h under shaking at 200 rpm. To allow biofilm formation, tubes were then incubated for 48 h under static conditions. Biofilm formation was evaluated following the methodology described by O’Toole et al. (19). Absorbance of the crystal violet-stained biofilms was measured at 550 nm.

**TABLE 2:** Environmental conditions evaluated for biofilm formation

Growth condition	Incubation temp (°C)^*a*^	pH	Phosphorus source
Control (MMS)	28	7	MMS phosphorus sources
Acidic pH	28	5.5	MMS phosphorus sources
Phosphorus fertilizer	28	7	Phosphate rock
High temperature	35	7	MMS phosphorus sources

a Temperature of incubation under static conditions.

### Metabolic-diversity assays.

The cellular masses of B02, L3, and Sp20, extracted from a culture grown in TY, were resuspended in a NaCl solution (0.85%). Cell pellets were recovered after 5 min of centrifugation at 6,000 rpm and resuspended in NaCl solution. This step was repeated twice to ensure that medium debris did not remain in the cell suspension. A total of 100 μl of the cell suspension adjusted to an OD (590 nm) of 0.1 was poured on 96 wells of Biolog Eco plates (Biolog Inc., CA). The OD (590 nm) in each well was measured at 12-h intervals and up to 72 hpi and then at 84 hpi using a Multiskan GO microplate spectrophotometer (Thermo Scientific).

### Statistical analysis.

Three replicates were carried out for each experiment. For analyses, halo areas were modeled as a square using *A* = *r*_1_ × *r*_2_. Data were log (*x* + 1) transformed to homogenize variances between treatments and normalize them. Linear models (LM) and linear mixed-effects models (LMM) were used for statistical analyses, using R v. 3.6.0 with packages lme4, car, and emmeans. Wald tests were used to establish the significance of the variables in the model. For significant variables, *post hoc* pairwise statistical testing was conducted for all models, using Tukey’s tests with degrees of freedom calculated with the Kenward-Roger approximation in the case of LMM. Normality and homoscedasticity assumptions were assessed for model validation by inspecting visually the histogram of the models’ residuals and using Levene’s tests of homogeneity of variance for each factor.

Phosphate solubilization data were analyzed with an LM including substrate, strain, and time as factors and the halo as a response variable. Pairwise comparisons were performed for levels of each factor and the interaction between levels of all factors. The differences in siderophore production between strains were analyzed with one-way analysis of variance (ANOVA) with strain as factor. An LMM was used for the analyses of surface motility. The model included bacteria and time as factors and halo as a response variable. Repeated measures over time on the same plate were considered in the model as a random effect. A similar LMM was used for surface spreading analysis but with time modeled as a continuous variable and including the interaction between bacteria and time. *Post hoc* testing was carried out for the interaction. Resistance to reactive oxygen species was analyzed with an LM using bacteria as a factor, H_2_O_2_ concentration as a continuous variable, and halo as a response variable. The differences in resistance were compared across strains. Biofilm formation was analyzed by means of an LM that included bacteria, treatment, and their interaction as categorical factors and absorbance as a response variable. *Post hoc* testing was performed for the interaction between the factors.

Finally, for the analysis of metabolic diversity, LMM were fit for OD as a function of time for the different strains on each well. The plate was established as a random effect to account for repeated measures over time. Statistically significant changes in OD over time were considered positive degradation velocity. Degradation velocities (model estimates for the slopes) were statistically compared across strains by using strain as a factor in the mixed-effect model and running a *post hoc* test on the time-strain interaction.

### MCDA.

A multicriteria decision analysis (MCDA) was carried out to exemplify decision-making based on phenotypic attributes for promising strain selection. MCDA methods provide a formal framework for standardized decision-making that allows the integration of different attributes for a comparative assessment in situations where there are multiple criteria involved. These methods are particularly suited for situations in which conflicting objectives for selection may arise, making tradeoffs commonly unavoidable (43). For example, an option could be the optimal under a set of defined criteria, but the same option could be the worst under a different set. For our analysis, the multiattribute utility theory (MAUT) approach was adopted using R v. 3.6.0 and with the package DecisionAnalysis. This methodology summarizes multiple attributes into a single quantitative score that can be compared across the different options available. Initially, we classified all the evaluated phenotypes (attributes) for each strain into three categories: plant growth enhancement, plant colonization stage, and ability to persist in the rhizosphere. These categories were defined based on the requirements that the active ingredient of a biofertilizer should have. The selected microorganisms should be able to enhance plant growth, colonize, and persist in the rhizosphere (1, 3). The phenotypic attributes measured in the study were assigned to each criterion (Table S3). For each of the phenotypic attributes, we established as performance values the adjusted means extracted from the statistical models employed for data analysis by using the package emmeans, except for carbon source degradation, for which degradation velocities were used (Fig. 4). Values were homogenized into scores of 0 to 1 using exponential single-value attribute functions. The function applied converts the performance value achieved for each attribute (phenotype in this case) into a number indicating how optimal is the performance for the decision maker. Maximum, minimum, and midpoint values for the construction of the functions, which determine the optimal values for each attribute, are provided in Table S3. As a next step in the analysis, scenarios of selection criteria were created based on difference preferences for the selection. Normalized weights, reflecting differences in importance, were assigned to the scores constructing three different scenarios (Table S3). The first scenario considered more relevant the phenotypes related to plant growth promotion, assigning a higher weight to criterion 1 and the same weight to criteria 2 and 3. In scenario 2, the same weight was assigned to plant growth and colonization traits and less weight was considered for persistence mechanisms. Scenario 3 assigned equal weights to all three criteria. Finally, for criterion aggregation, the total score for each bacterium and scenario was computed as a linear weighted sum of scores across attributes (49). Hierarchical structure and interactions between attributes were not considered for the analysis. For the sake of simplicity, sensitivity analysis was not included in the MCDA.

### Data availability.

The resulting 16S rRNA gene sequences of B02, L3, and Sp20 were deposited in the GenBank database of the National Center for Biotechnology Information (NCBI [https://www.ncbi.nlm.nih.gov]) under accession numbers MK559029, MK559030, and MK559031, respectively.

## Supplementary Material

Supplemental file 1
