# Supplementary material for: A Framework for the Selection of Plant Growth-Promoting Rhizobacteria Based on Bacterial Competence Mechanisms
Source: Appl Environ Microbiol. 2020 Jul 2;86(14):e00760-20. doi: 10.1128/AEM.00760-20 (PMC7357491; doi:10.1128/AEM.00760-20)
Supplement: Supplemental file 1 [file AEM.00760-20-s0001.pdf]

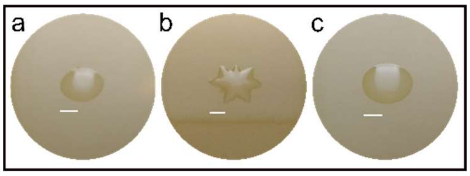


Figure S1. Surface spreading colonies of B02 (a), L3 (b) and Sp20 (c) at 48 hours post inoculation on semi-solid modified MM (MMS). Bars, 0.5 cm.

Table S1. Media composition for the photometric phosphate test

| Media components | Concentration (g/L) | | | | |
|---|---|---|---|---|---|
| | Mnn/Ye | Mnn/$NH_4$ | Glc/$NH_4$ | Suc/$NH_4$ | Frt/$NH_4$ |
| Mannitol | 10 | 10 | | | |
| Sucrose | | | 10 | | |
| Glucose | | | | 10 | |
| Fructose | | | | | 10 |
| Yeast extract | 0.5 | | | | |
| $NH_4SO_4$ | | 0.5 | 0.5 | 0.5 | 0.5 |
| $MgSO_4$. $7H_2O$ | 0.2 | 0.2 | 0.2 | 0.2 | 0.2 |
| NaCl | 0.1 | 0.1 | 0.1 | 0.1 | 0.1 |
| Phosphate rock | 5 | 5 | 5 | 5 | 5 |

Mnn/ye (mannitol/yeast extract), Mnn/$NH_4$ (mannitol/$NH_4SO_4$), Glc/$NH_4$ (Glucose/$NH_4SO_4$), Suc/$NH_4$ (Sucrose/$NH_4SO_4$) and Frt/$NH_4$ (Fructose/$NH_4SO_4$).

Table S2. Final viability (Ln CFU/mL) and pH

| Measured factor | Mnn/Ye | Mnn/$NH_4$ | Glc/NH4 | Suc/NH4 | Frt/$NH_4$ |
|---|---|---|---|---|---|
| Ln CFU/mL B02 | 24.3 ± 0.046 | 24.1 ± 0.17 | 24.5 ± 0.035 | 23.6 ± 0.061 | 23.9 ± 0.075 |
| Ln CFU/mL L3 | 22.9 ± 0.029 | 22.3 ± 0.079 | 22.2 ± 0.022 | 22.1 ± 0.056 | 20.1 ± 0.073 |
| Ln CFU/mL Sp20 | 24.4 ± 0.052 | 23.0 ± 0.033 | 23.1 ± 0.16 | 24.6 ± 0.11 | 23.6 ± 0.19 |
| pH B02 | 6.67 ± 0.01 | 4.15 ± 0.014 | 4.28 ± 0.008 | 4.28 ± 0.021 | 4.3 ± 0.011 |
| pH L3 | 6.69 ± 0.005 | 4.41 ± 0.013 | 4.34 ±0.011 | 4.41 ±0.011 | 4.61 ± 0.014 |
| pH Sp20 | 6.86 ± 0.021 | 4.17 ± 0.015 | 4.39 ± 0.017 | 4.15 ± 0.014 | 4.22 ± 0.016 |

*average value ± TE. Colony forming units (CFU)

Table S3. Multicriteria decision analysis weighted scores assigned to the bacterial traits evaluated for B02, L3 and Sp20.

| | | | | | | | | | | Scenario 1 score | | | | | Scenario 2 score | | | | | Scenario 3 score | | | | |
|---|---|---|---|---|---|---|---|---|---|---|---|---|---|---|---|---|---|---|---|---|---|---|---|---|
| Group | Associated phenotypes | Abbreviation | Culture conditions | Lowest | Midpoint | Highest | B02 MAVF Score | L3 MAVF Score | Sp20 MAVF Score | Group Weight | Weight | B02 Weighted SAVF | L3 Weighted SAVF | Sp20 Weighted SAVF | Group weight | Weight | B02 Weighted SAVF | L3 Weighted SAVF | Sp20 Weighted SAVF | Group weight | Weight | B02 Weighted SAVF | L3 Weighted SAVF | Sp20 Weighted SAVF |
| | | Pca | NBRIP agar + $Ca_3PO_4$ | 0 | 3.49 | 50.27 | 0.41 | 0.54 | 0.54 | | 0.03 | 0.014 | 0.018 | 0.018 | | 0,025 | 0,010 | 0,013 | 0,013 | | 0,039 | 0,016 | 0,021 | 0,021 |
| | | Pfe | NBRIP agar + $FePO_4$ | 0 | 0.46 | 50.27 | 0.47 | 0.54 | 0.48 | | 0.03 | 0.016 | 0.018 | 0.016 | | 0,025 | 0,012 | 0,013 | 0,012 | | 0,039 | 0,019 | 0,021 | 0,019 |
| | | Prk | NBRIP agar + rock phosphate | 0 | 0.74 | 50.27 | 0.50 | 0.51 | 0.49 | | 0.03 | 0.017 | 0.017 | 0.016 | | 0,025 | 0,012 | 0,013 | 0,012 | | 0,039 | 0,020 | 0,020 | 0,019 |
| | | PMY | NBRIP broth + mannitol + yeast extract | 0 | 4.47 | 100 | 0.55 | 0.39 | 0.55 | | 0 | 0 | 0 | 0 | | 0,000 | 0,000 | 0,000 | 0,000 | | 0,000 | 0,000 | 0,000 | 0,000 |
| Plant growth promotion | Phosphate solubilization | PMN | NBRIP broth + mannitol + $NH_4SO_4$ | 0 | 63.23 | 100 | 0.61 | 0.37 | 0.53 | 0.57 | 0.1 | 0.061 | 0.037 | 0.053 | 0.425 | 0,074 | 0,045 | 0,028 | 0,039 | 0.333 | 0,045 | 0,027 | 0,017 | 0,024 |
| | | PGN | NBRIP broth + glucose + $NH_4SO_4$ | 0 | 56.42 | 100 | 0.49 | 0.47 | 0.54 | | 0.1 | 0.049 | 0.047 | 0.054 | | 0,074 | 0,036 | 0,035 | 0,040 | | 0,045 | 0,022 | 0,021 | 0,024 |
| | | PSN | NBRIP broth + sucrose + $NH_4SO_4$ | 0 | 57.23 | 100 | 0.51 | 0.48 | 0.51 | | 0.1 | 0.051 | 0.048 | 0.051 | | 0,074 | 0,038 | 0,035 | 0,038 | | 0,045 | 0,023 | 0,022 | 0,023 |
| | | PFN | NBRIP broth + fructose + $NH_4SO_4$ | 0 | 36.71 | 100 | 0.55 | 0.34 | 0.59 | | 0.1 | 0.055 | 0.034 | 0.059 | | 0,074 | 0,041 | 0,025 | 0,043 | | 0,045 | 0,025 | 0,015 | 0,026 |
| | Siderophores production | Sph | Iron deficient medium | 0 | 73.9 | 100 | 0.70 | 0.74 | 0.33 | | 0.07 | 0.049 | 0.052 | 0.023 | | 0,055 | 0,038 | 0,041 | 0,018 | | 0,035 | 0,024 | 0,026 | 0,011 |
| | Swimming | Sw | Bromfield media + 0.3 % agar | 0 | 2.13 | 50.27 | 0.51 | 0.47 | 0.51 | | 0.07 | 0.036 | 0.033 | 0.036 | | 0,106 | 0,054 | 0,050 | 0,055 | | 0,083 | 0,043 | 0,039 | 0,043 |
| Colonization | Surface spreading | Ss | Modified minimal medium + 0.6 % agar | 0 | 0.71 | 50.27 | 0.47 | 0.60 | 0.42 | 0.28 | 0.07 | 0.033 | 0.042 | 0.030 | 0.425 | 0,106 | 0,049 | 0,063 | 0,045 | 0.333 | 0,083 | 0,039 | 0,050 | 0,035 |
| | ROS scavenging | ros | Minimal medium | 0 | 0.87 | 50.27 | 0.66 | 0.47 | 0.40 | | 0.07 | 0.046 | 0.033 | 0.028 | | 0,106 | 0,070 | 0,050 | 0,043 | | 0,083 | 0,055 | 0,039 | 0,034 |
| | Biofilm formation | BfM | Modified minimal medium | 0 | 0.51 | 1 | 0.45 | 0.52 | 0.53 | | 0.07 | 0.032 | 0.036 | 0.037 | | 0,106 | 0,048 | 0,055 | 0,056 | | 0,083 | 0,038 | 0,043 | 0,044 |
| | | BfpH | Modified minimal medium (acidic pH) | 0 | 0.22 | 1 | 0.51 | 0.49 | 0.51 | | 0.03 | 0.015 | 0.015 | 0.015 | | 0,030 | 0,015 | 0,015 | 0,015 | | 0,067 | 0,034 | 0,033 | 0,034 |
| | Biofilm formation under stress conditions | BfT | Modified minimal medium (High temperature) | 0 | 0.23 | 1 | 0.48 | 0.52 | 0.50 | | 0.03 | 0.015 | 0.015 | 0.015 | | 0,030 | 0,015 | 0,015 | 0,015 | | 0,067 | 0,032 | 0,034 | 0,033 |
| | | Bfrk | Modified minimal medium + rock phosphate | 0 | 0.39 | 1 | 0.25 | 0.68 | 0.53 | | 0.03 | 0.008 | 0.020 | 0.016 | | 0,030 | 0,008 | 0,020 | 0,016 | | 0,067 | 0,017 | 0,045 | 0,035 |
| | | 1 | α-D-lactose | 0 | 0.016 | 0.028 | 0.07 | 0.79 | 0.64 | | 0.003 | 0.000 | 0.002 | 0.002 | | 0,003 | 0,000 | 0,002 | 0,002 | | 0,006 | 0,000 | 0,005 | 0,004 |
| Persistence | | 2 | β-methyl-d-glucoside | 0 | 0.016 | 0.028 | 0.47 | 0.74 | 0.58 | 0.150 | 0.003 | 0.001 | 0.002 | 0.002 | 0.150 | 0,003 | 0,001 | 0,002 | 0,002 | 0.333 | 0,006 | 0,003 | 0,005 | 0,003 |
| | | 3 | D-cellobiose | 0 | 0.016 | 0.028 | 0.69 | 0.67 | 0.35 | | 0.003 | 0.002 | 0.002 | 0.001 | | 0,003 | 0,002 | 0,002 | 0,001 | | 0,006 | 0,004 | 0,004 | 0,002 |
| | Metabolic diversity | 4 | D-galactonic acid γ-lactone | 0 | 0.016 | 0.028 | 0.85 | 0.79 | 0.28 | | 0.003 | 0.002 | 0.002 | 0.001 | | 0,003 | 0,002 | 0,002 | 0,001 | | 0,006 | 0,005 | 0,005 | 0,002 |
| | | 6 | i-Erythritol | 0 | 0.016 | 0.028 | 0.00 | 0.01 | 0.00 | | 0.003 | 0.000 | 0.000 | 0.000 | | 0,003 | 0,000 | 0,000 | 0,000 | | 0,006 | 0,000 | 0,000 | 0,000 |
| | | 7 | Glucose-1-phosphate | 0 | 0.016 | 0.028 | 0.83 | 0.83 | 0.48 | | 0.003 | 0.002 | 0.002 | 0.001 | | 0,003 | 0,002 | 0,002 | 0,001 | | 0,006 | 0,005 | 0,005 | 0,003 |
| | | 8 | D-mannitol | 0 | 0.016 | 0.028 | 0.82 | 0.85 | 0.80 | | 0.003 | 0.002 | 0.002 | 0.002 | | 0,003 | 0,002 | 0,002 | 0,002 | | 0,006 | 0,005 | 0,005 | 0,005 |

| | | | | | | | | | | | | | | | | | | | |
|---|---|---|---|---|---|---|---|---|---|---|---|---|---|---|---|---|---|---|---|
| 9 | N-acetyl-d-glucosamine | 0 | 0.016 | 0.028 | 0.54 | 0.63 | 0.53 | 0.003 | 0.001 | 0.002 | 0.001 | 0,003 | 0,001 | 0,002 | 0,001 | 0,006 | 0,003 | 0,004 | 0,003 |
| 10 | D-Xylose | 0 | 0.016 | 0.028 | 0.89 | 0.70 | 0.70 | 0.003 | 0.002 | 0,002 | 0,002 | 0,003 | 0,002 | 0,002 | 0,002 | 0,006 | 0,005 | 0,004 | 0,004 |
| 12 | 4-Hydroxybenzoic acid | 0 | 0.016 | 0.028 | 0.31 | 0.42 | 0.09 | 0.003 | 0.001 | 0,001 | 0,000 | 0,003 | 0,001 | 0,001 | 0,000 | 0,006 | 0,002 | 0,003 | 0,001 |
| 13 | α-Ketobutyric acid | 0 | 0.016 | 0.028 | 0.00 | 0.05 | 0.05 | 0.003 | 0.000 | 0,000 | 0,000 | 0,003 | 0,000 | 0,000 | 0,000 | 0,006 | 0,000 | 0,000 | 0,000 |
| 14 | D-Galacturonic acid | 0 | 0.016 | 0.028 | 0.72 | 0.44 | 0.00 | 0.003 | 0.002 | 0,001 | 0,000 | 0,003 | 0,002 | 0,001 | 0,000 | 0,006 | 0,004 | 0,003 | 0,000 |
| 15 | D-Glucosaminic acid | 0 | 0.016 | 0.028 | 0.09 | 0.00 | 0.00 | 0.003 | 0.000 | 0,000 | 0,000 | 0,003 | 0,000 | 0,000 | 0,000 | 0,006 | 0,001 | 0,000 | 0,000 |
| 16 | D-Malic acid | 0 | 0.016 | 0.028 | 0.09 | 0.56 | 0.00 | 0.003 | 0.000 | 0,002 | 0,000 | 0,003 | 0,000 | 0,002 | 0,000 | 0,006 | 0,001 | 0,003 | 0,000 |
| 17 | γ-Hydroxybutyric acid | 0 | 0.016 | 0.028 | 0.02 | 0.48 | 0.03 | 0.003 | 0.000 | 0,001 | 0,000 | 0,003 | 0,000 | 0,001 | 0,000 | 0,006 | 0,000 | 0,003 | 0,000 |
| 19 | Pyruvic acid methyl ester | 0 | 0.016 | 0.028 | 0.49 | 0.65 | 0.42 | 0.003 | 0.001 | 0,002 | 0,001 | 0,003 | 0,001 | 0,002 | 0,001 | 0,006 | 0,003 | 0,004 | 0,003 |
| 25 | Tween 80 | 0 | 0.016 | 0.028 | 0.00 | 0.03 | 0.00 | 0.003 | 0.000 | 0,000 | 0,000 | 0,003 | 0,000 | 0,000 | 0,000 | 0,006 | 0,000 | 0,000 | 0,000 |
| 26 | Glycyl-l-glutamic acid | 0 | 0.016 | 0.028 | 0.89 | 0.45 | 0.00 | 0.003 | 0.002 | 0,001 | 0,000 | 0,003 | 0,002 | 0,001 | 0,000 | 0,006 | 0,005 | 0,003 | 0,000 |
| 27 | L-Arginine | 0 | 0.016 | 0.028 | 0.78 | 0.51 | 0.42 | 0.003 | 0.002 | 0,001 | 0,001 | 0,003 | 0,002 | 0,001 | 0,001 | 0,006 | 0,005 | 0,003 | 0,003 |
| 28 | L-Asparagine | 0 | 0.016 | 0.028 | 0.82 | 0.91 | 0.70 | 0.003 | 0.002 | 0,002 | 0,002 | 0,003 | 0,002 | 0,002 | 0,002 | 0,006 | 0,005 | 0,006 | 0,004 |
| 30 | L-Serine | 0 | 0.016 | 0.028 | 1.00 | 0.44 | 0.67 | 0.003 | 0.003 | 0,001 | 0,002 | 0,003 | 0,003 | 0,001 | 0,002 | 0,006 | 0,006 | 0,003 | 0,004 |
| 31 | L-Threonine | 0 | 0.016 | 0.028 | 0.57 | 0.29 | 0.38 | 0.003 | 0.002 | 0,001 | 0,001 | 0,003 | 0,002 | 0,001 | 0,001 | 0,006 | 0,003 | 0,002 | 0,002 |

The abbreviations used for each attribute are the following: Phosphate solubilization in NBRIP containing $Ca_3PO_4$ (Pca), FePO4 (Pfe), rock phosphate (Prk), mannitol and yeast extract (PMY), $NH_4SO_4$ and mannitol (PMN), glucose (PGN), sucrose (PSN) or fructose (PFN). Siderophores synthesis (Sph), swimming (Sw), surface spreading (Ss), ROS sensibility (ros). Biofilm formation in modified minimal medium (BfM), at acidic pH (BfpH), at high temperature (BfT) and in presence of rock phosphate (Bfrk). Utilization of α-D-lactose (1), β-methyl-d-glucoside (2), D-cellobiose (3), D-galactonic acid γ-lactone (4), i-Erythritol (6), Glucose-1-phosphate (7), D-mannitol (8), N-acetyl-d-glucosamine (9), D-Xylose (10), 4-Hydroxybenzoic acid (12), α-Ketobutyric acid (13), D-Galacturonic acid (14), D-Glucosaminic acid (15), D-Malic acid (16), γ-Hydroxybutyric acid (17), Pyruvic acid methyl ester (19), Tween 80 (25), Glycyl-l-glutamic acid (26), L-Arginine (27), L-Asparagine (28), L-Serine (30), L-Threonine (31).
